# Deferoxamine Improves Radiation‐Induced Peripheral Neuropathy

**DOI:** 10.1111/jcmm.71227

**Published:** 2026-06-07

**Authors:** Christopher V. Lavin, Alexander Z. Fazilat, Carter B. Kendig, Palca Shibale, Kelly X. Huang, Sriya Nemani, Jennifer B. Parker, Caleb Valencia, Naga A. R. Ailury, Michelle Griffin, Arash Momeni, Michael T. Longaker, Derrick C. Wan

**Affiliations:** ^1^ Hagey Laboratory for Pediatric Regenerative Medicine Stanford University School of Medicine Stanford California USA; ^2^ Department of Surgery, Division of Plastic and Reconstructive Surgery Stanford University School of Medicine Stanford California USA

**Keywords:** demyelination, ferroptosis, fibrosis, nerve injury, peripheral nerve, radiation, radiotherapy, reactive oxygen species, regeneration

## Abstract

Radiation‐induced peripheral neuropathy (RIPN) is a devastating sequela of radiation therapy (XRT). Current treatment options are limited. Deferoxamine (DFO) has been useful in treating radiation‐induced dermal fibrosis. This study aimed to evaluate DFO for RIPN. Thus, 18 mice received 30Gy of fractionated XRT. After a fibrosis development interval, mice were treated with DFO injections, saline injections (Saline) or none (IR) (*n* = 6 per group). Longitudinal measures included footprint analysis, cold allodynia testing and monofilament testing. Immunofluorescent staining for myelination (MPZ) and axonal regeneration (GAP43) took place at the conclusion of the experiment. DFO improved motor deficits (Combined Toe Spread scores: −9.72, −12.91, −12.72 for DFO, Saline and IR, respectively). Additionally, DFO improved cold allodynia (duration ratios: 0.90, 0.70 and 0.73 for DFO, Saline and IR, respectively). Monofilament testing revealed the same trend, though not statistically significant. Additionally, DFO increased remyelination on MPZ staining (normalized myelin ratios: 0.84 DFO, 0.74 Saline, 0.72 IR) and increased axonal regeneration on GAP43 staining compared to all groups (pixel area: 3.76% DFO, 1.95% Saline, 1.94% IR, 2.09% Control). In conclusion, this murine study revealed DFO improves RIPN sensorimotor function. This is encouraging as disease‐modifying treatments are limited for patients suffering from this XRT side effect.

## 1 Introduction

Radiation‐induced peripheral neuropathy (RIPN) is a progressive, debilitating disease process that can result as an unfortunate side effect of radiation therapy (XRT). Although less common than other side effects, if present, RIPN causes a massive detriment to quality of life for patients, oftentimes causing pain or possibly affecting both motor and sensory function. Correlating with higher dose XRT regimens (> 50 Gy), there is a reported 1%–19% incidence of RIPN in the literature with modern fractionated regimens [1, 2, 3, 4, 5, 6, 7, 8]. RIPN is commonly described in the brachial and lumbar plexuses following breast/head/neck or pelvic radiation exposure, respectively; however, it can affect any area of the body where XRT has taken place, including the cranial nerves. Its incidence, presentation and severity are highly variable, making it difficult to predict and study. Additionally, it may present many years after XRT and be confused with comorbidities such as diabetic/vascular neuropathies or chemotherapy neurotoxicity [5, 9, 10]. Seeing more cases of clinically significant, chronic RIPN may be a function of cancer survivors living longer due to advancements in oncologic treatment and be more common than initially expected, but nevertheless creates a significant problem with minimal treatments available. The noninvasive therapies that do currently exist are limited to symptomatic management and avoidance of further insult [11, 12, 13].

Ionizing radiation enacts its antitumor effects via direct DNA damage, inducing apoptosis and other cell death pathways. This inevitably injures all tissues in the irradiated field, though disproportionately affects rapidly dividing and highly metabolic cells. Historically, peripheral nerves were thought to be spared from radiation injury as they are less rapidly dividing, however, this fallacy was likely a product of follow‐up timelines that were too short and a focus on survival over symptoms. XRT generates reactive oxygen species (ROS), and through a destructive cascade of inflammatory events, microvascular injury and hypoperfusion, further induces cell death pathways and perpetuates a cyclical feedback loop of inflammation. This leads to fibroblast recruitment and a prolonged, aberrant healing response which culminates in disorganized extracellular matrix production and hypocellular tissues. This diffuse fibroatrophic scar‐like landscape is referred to as radiation‐induced fibrosis (RIF) [14]. It is thought that chronic RIPN follows a similar trajectory [15]. Injury occurs directly at the nerve level, and importantly, in the surrounding soft tissues as well. In many cases, particularly in plexopathy literature, there exists some level of compression on the nerves secondary to the surrounding soft tissue attenuation and RIF [7, 16, 17]. This compression further diminishes nerve blood flow, leading to symptoms. A commonly used analogy for this compressive phenomenon is carpal tunnel syndrome, in which inflammation/expansion of contents in the tunnel created by the carpal bones and flexor retinaculum in the palm restricts blood flow to the median nerve, generating well‐known numbness and tingling symptoms [18].

Deferoxamine (DFO) holds promise as an effective treatment for RIF as it addresses two primary components of its pathogenesis [19]. Local delivery of DFO acts to chelate free ferric iron in the tissues. This removes the necessary catalyst for ROS‐producing Fenton reactions, limiting the self‐perpetuating cycle of ROS‐mediated chronic inflammation and ferroptosis in the subacute and chronic phases of radiation injury. Additionally, the degradation of transcription factor hypoxia‐inducible factor 1α (HIF‐1α) is dependent on iron as a cofactor. DFO's iron chelation acts to stabilize HIF‐1α and upregulates angiogenesis and neovascularization in tissues, providing improved blood flow and thus oxygen and nutrient delivery to RIF‐afflicted tissues. Several preclinical and early clinical studies from our group have investigated DFO for dermal RIF, finding success in reversing and partially preventing skin damage [20, 21, 22, 23]. As it pertains to nerve injury, however, DFO studies have been primarily focused on central nervous system injury or small animal peripheral nerve compression/transection injury models [24, 25, 26, 27, 28, 29]. These reveal DFO's utility as a protective medication and nerve regeneration enhancer following these insults. Therefore, we designed a murine study to determine DFO's potential for the treatment of RIPN as well.

## 2 Methods

### 2.1 Animals

Eighteen C57BL/6 mice (The Jackson Laboratory, Bar Harbour, ME, USA) aged 12 weeks underwent right hindlimb irradiation. Mice were divided into 3 groups (*n* = 6 each): irradiation only control (IR group), deferoxamine injections (DFO group) or sterile saline injections (Saline group). Equal numbers of male and female mice were included in each group to control for any sex differences. All experimentation adhered to Stanford University's Institutional Animal Care and Use Committee guidelines and under an established protocol (IACUC 11048) in accordance with National Institutes of Health guidelines.

### 2.2 Irradiation

**FIGURE 1:**
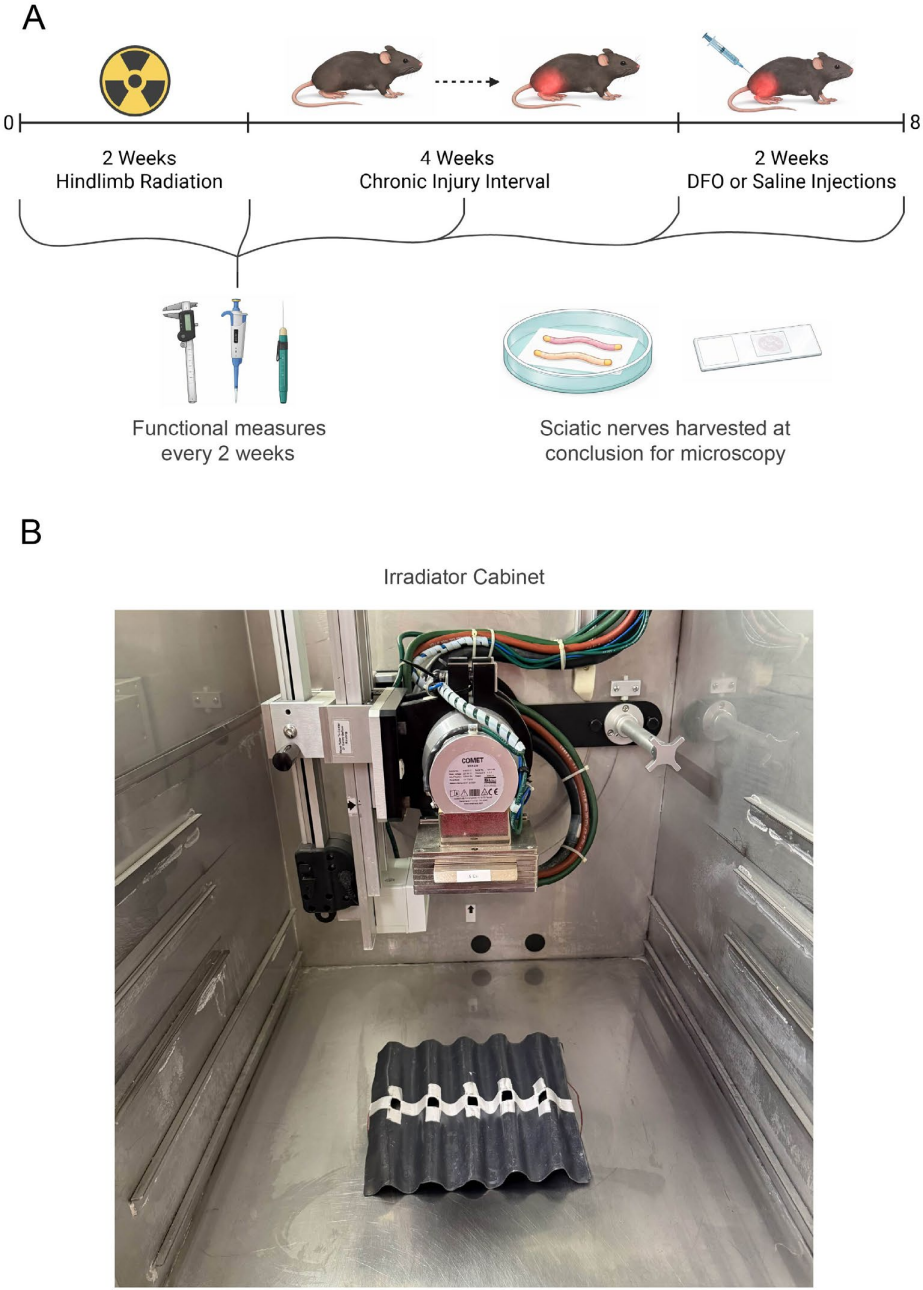
Irradiation and treatment timeline. (A) Timeline: 2‐week irradiation period, 4‐week fibrosis development interval, 2‐week treatment period with deferoxamine (DFO) or saline. (B) Irradiation device: Kimtron Polaris SC‐500 x‐ray machine (Kimtron Inc., Oxford, CT, USA). Mice were anaesthetised and placed under lead shielding to protect all but their right hindlegs during their six sessions of 5 Gy (30 Gy total over 2 weeks).

Using a Kimtron Polaris SC‐500 x‐ray machine (Kimtron Inc., Oxford, CT, USA), right hind legs received 30 Gy of radiation, fractionated into six 5 Gy sessions every other day for 12 days [20] (Figure 1A). Mice were anaesthetised with weight‐based ketamine and xylazine dosing to remain still in the irradiator. Lead shielding was used to protect the rest of each mouse's body (Figure 1B). A 4‐week injury development interval, consistent with other reports on chronic RIF injury, was allowed to pass before proceeding with the DFO or Saline injections [20].

### 2.3 Treatments

Deferoxamine mesylate salt (Sigma‐Aldrich, St. Louis, MO, USA) was dissolved into sterile saline at a concentration of 30 mg/1 mL. DFO mice received peri‐sciatic intramuscular injections of 3 mg DFO (100 μL of solution) every other day for 2 weeks after the RIF development interval. Saline mice received 100 μL of sterile saline alone over the same period. Injections were given under 2% isoflurane inhaled anaesthesia and administered slowly with care taken to avoid direct sciatic nerve trauma, monitoring for muscular twitch.

### 2.4 Functional Measures

**FIGURE 2:**
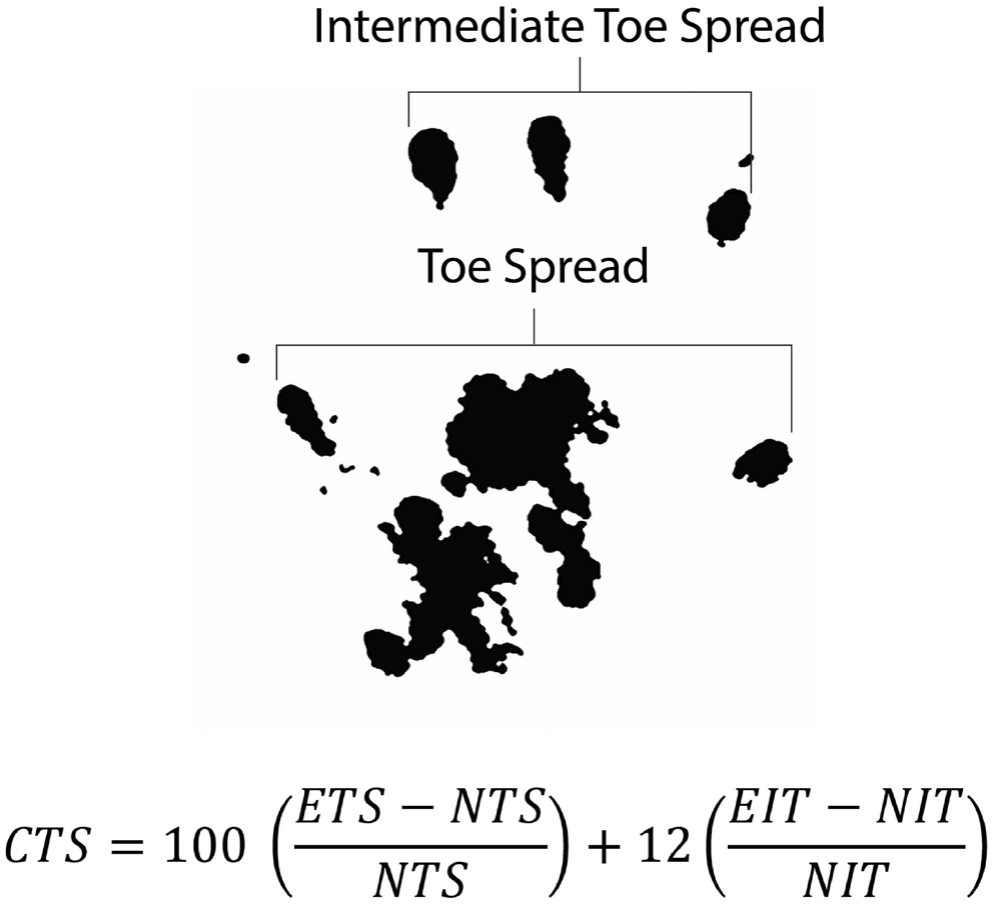
Combined Toe Spread measurement and formula. **Mouse hind paws were inked and measured to determine changes in motor function. As rat Sciatic Functional Index (SFI) is less reliable in mice, Combined Toe Spread was calculated. (ETS, Experimental Toe Spread (Right digits 1–5 spread), NTS, Normal Toe Spread (Left digits 1–5 spread), EIT, Experimental Intermediate Toe Spread (Right digits 2–4 spread), NIT, Normal Intermediate Toe Spread (Left digits 2–4 spread)).

**FIGURE 3:**
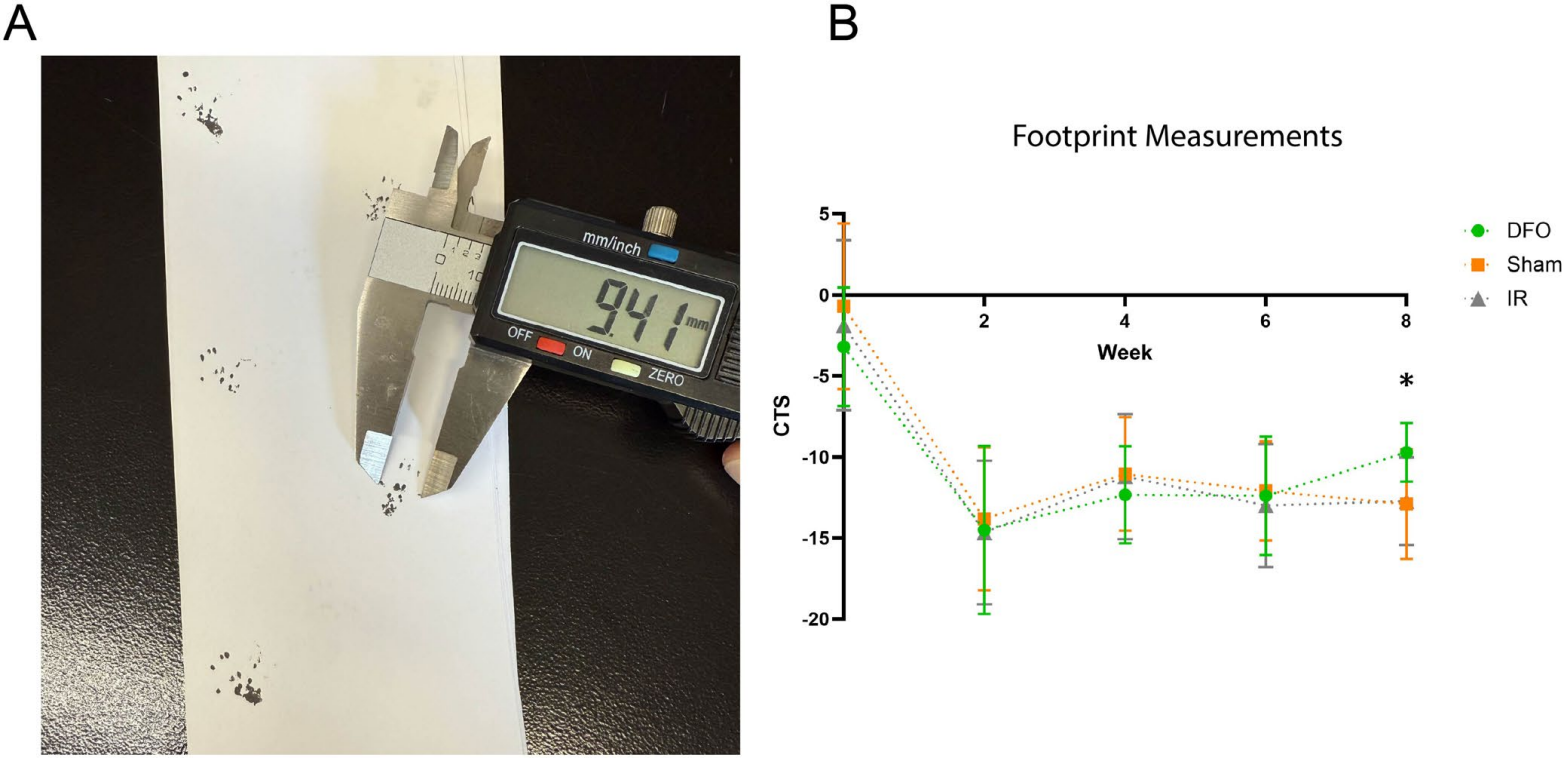
Footprint measurement and analysis. (A) Manual measurements of footprint toe spreads and (B) Combined Toe Spread (CTS) comparison. No significant differences between groups were measured until after 2 weeks of treatment, at which time deferoxamine (DFO) injections improved normalized scores relative to saline injected and irradiated control (IR) groups. (−9.72, −12.91, −12.72 for DFO, Saline and IR, respectively (*p = 0.0469 for DFO vs. Saline and *p = 0.0481 for DFO vs. IR)). A score of 0 reflects normal toe spread, with more negative scores indicating greater functional deficit.

All sciatic nerve functional metrics were tracked longitudinally, starting prior to irradiation and every 2 weeks until the conclusion of the experiment. Footprint analysis was conducted by coating the plantar surfaces of both hind feet with black stamp ink. The mice were then placed in a thin, straight track lined with white paper and allowed to traverse the track to return to their cage. High precision callipers were used to measure the entire footprint width, designated as Toe Spread (TS), as well as the distance between digits 2–4 or Intermediate Toe Spread (IT) (Figure 2A). These values decrease with sciatic motor neuron injury as spreading of the toes is natural during normal gait and reliant on active digit abduction. This occurs at both the intrinsic level (interossei and lumbrical muscles) as well as extrinsically (flexor/extensor balance), as both the tibial and peroneal nerves are affected. Because of infrequent heel contact with the paper, paw length could not be reliably measured in many of the footprints. Therefore, the classic Sciatic Functional Index (SFI) used in the original rat studies could not be calculated, which is a common problem among mouse studies [30, 31, 32]. Instead, a simplified implementation of that formula which ignores paw length was used and designated Combined Toe Spread (CTS) (Figure 2B). Similar regression coefficients seen in the SFI formula were assigned to differentially weight the outcome more heavily in favour of TS over IT, 100 and 12 respectively. These coefficients were chosen to preserve the parameters' relative weighting in prior SFI formulations, and like the original paper, symmetric function clusters near 0, with more negative values indicating greater degrees of functional deficit. Normal left footprints TSs and ITs were averaged on each paper strip to be used in the CTS formula as baseline uninjured means for each individually tested right footprint to be compared to, generating several CTSs indexed against that strip's averaged left foot values (Figure 3A). Thus, each strip provided as many data points as there were radiation‐injured right footprints. Paper strips contained 2–5 footprints per foot, and if 2 or more footprints per side were not achieved, the mouse was re‐inked and a new strip was collected. CTS means were compared between groups at each time point.

**FIGURE 4:**
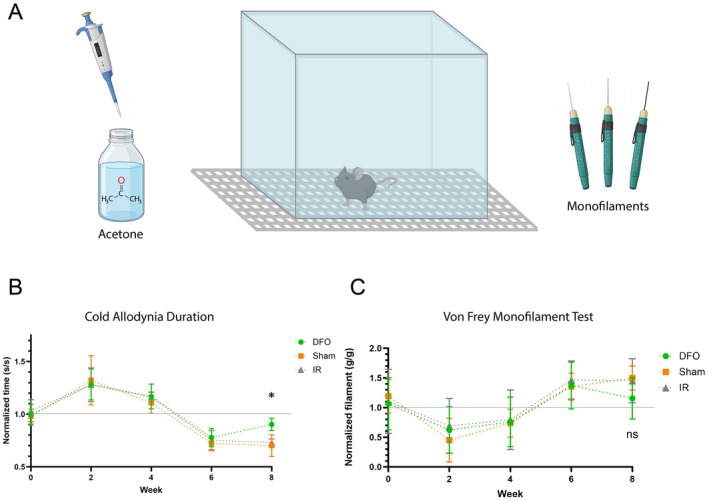
Sensory testing and analysis. (A) Mesh wire‐rack floor for mouse hind paw sensation testing. Cold allodynia duration measurement with acetone application, and Von Frey mechanical allodynia testing with custom weight monofilaments were performed after 30 min acclimatization periods. (B) Plantar acetone application for cold allodynia duration revealed no differences between groups until treatment. DFO treated hindlegs were less hyposensitive in the chronic phase of radiation‐induced peripheral nerve injury compared to Saline and IR groups. (0.90, 0.70 and 0.73 for DFO, Saline and IR, respectively (*p = 0.0179 for DFO vs. Saline and *p = 0.0480 for DFO vs. IR)). A score greater than 1 indicates hypersensitivity and less than 1 reflects hyposensitivity. (C) Von Frey mechanical allodynia testing confirmed hyposensitivity at the conclusion of the experiment, with DFO treatment reducing the degree of hyposensitivity compared to Saline and IR groups, though the difference was not statistically significant. (1.16 for DFO, 1.50 for Saline and 1.45 for IR (ns *p* = 0.2390 for DFO vs. Saline and ns *p* = 0.3323 for DFO vs. IR)). Normalized ratios less than 1 reflect hypersensitivity as lighter weight filaments elicit a pain withdrawal response, while ratios greater than 1 indicate hyposensitivity since heavier filaments were required.

To assess changes in sensory function, two tests were employed after mice were placed in a wire mesh floor cage for 30 min to acclimate (Figure 4A). Plantar application of 10 μL of acetone was performed, one foot at a time, and the duration of behaviour indicative of allodynia (shaking/twitching, limping, licking) due to the evaporation's cold sensation was recorded for each foot [33]. Right foot allodynia duration was normalized to that mouse's unaffected left foot, and means between groups were compared.

Mechanical sensitivity or manual Von Frey testing, was also performed with custom made monofilaments created from nylon sutures of varying sizes and lengths ranging from 0.01 g to 6 g, calibrated on a precision milligram scale [33]. Increasing filament weight was applied to the plantar surface of each mouse's feet from below the wire cage, until a sufficient filament elicited a withdrawal/flinch response. Again, right foot filament weight was normalized to the mouse's unaffected left foot, and means were compared between groups.

### 2.5 Immunofluorescence

**FIGURE 5:**
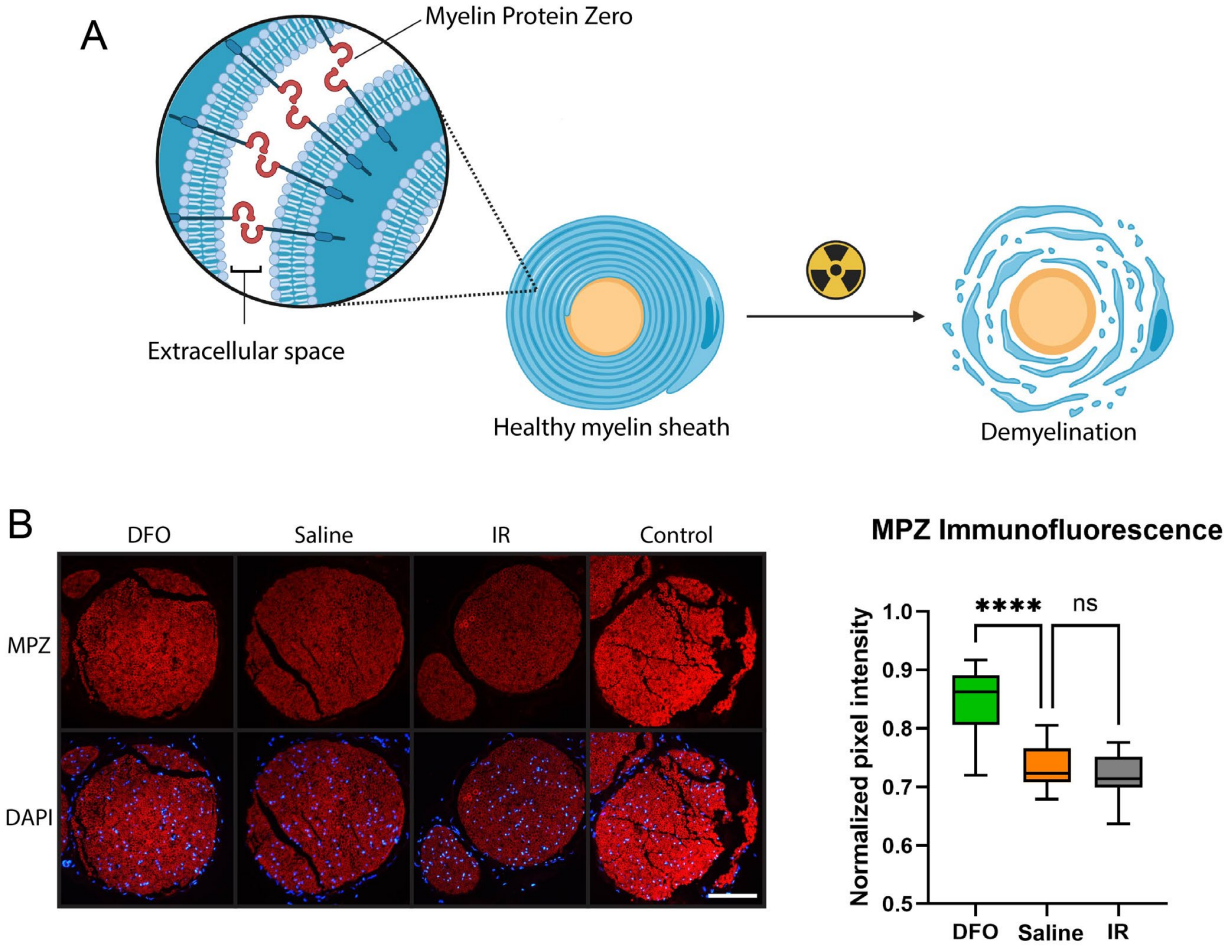
Myelin Protein Zero function and immunofluorescence. (A) Myelin Protein Zero (MPZ) is a transmembrane adhesion protein with an immunoglobulin‐like extracellular component that serves to form and stabilize the many lamellae of the Schwann cell myelin sheath. Given its structural importance, it serves as a proxy for healthy vs. demyelinated axons. (B) MPZ immunofluorescent staining revealed demyelination in all irradiated sciatic nerves, with deferoxamine (DFO) treatment reducing the deficit compared to saline and irradiated control (IR) groups. Results are displayed as normalized ratios against contralateral healthy hindlimbs. (0.84 for DFO, 0.74 for Saline and 0.72 for IR (****p < 0.0001 for DFO vs. Saline and DFO vs. IR)). Microscopy scale bar represents 100 μm. Box and whisker plots with line at medians, whiskers at minimum and maximum values.

**FIGURE 6:**
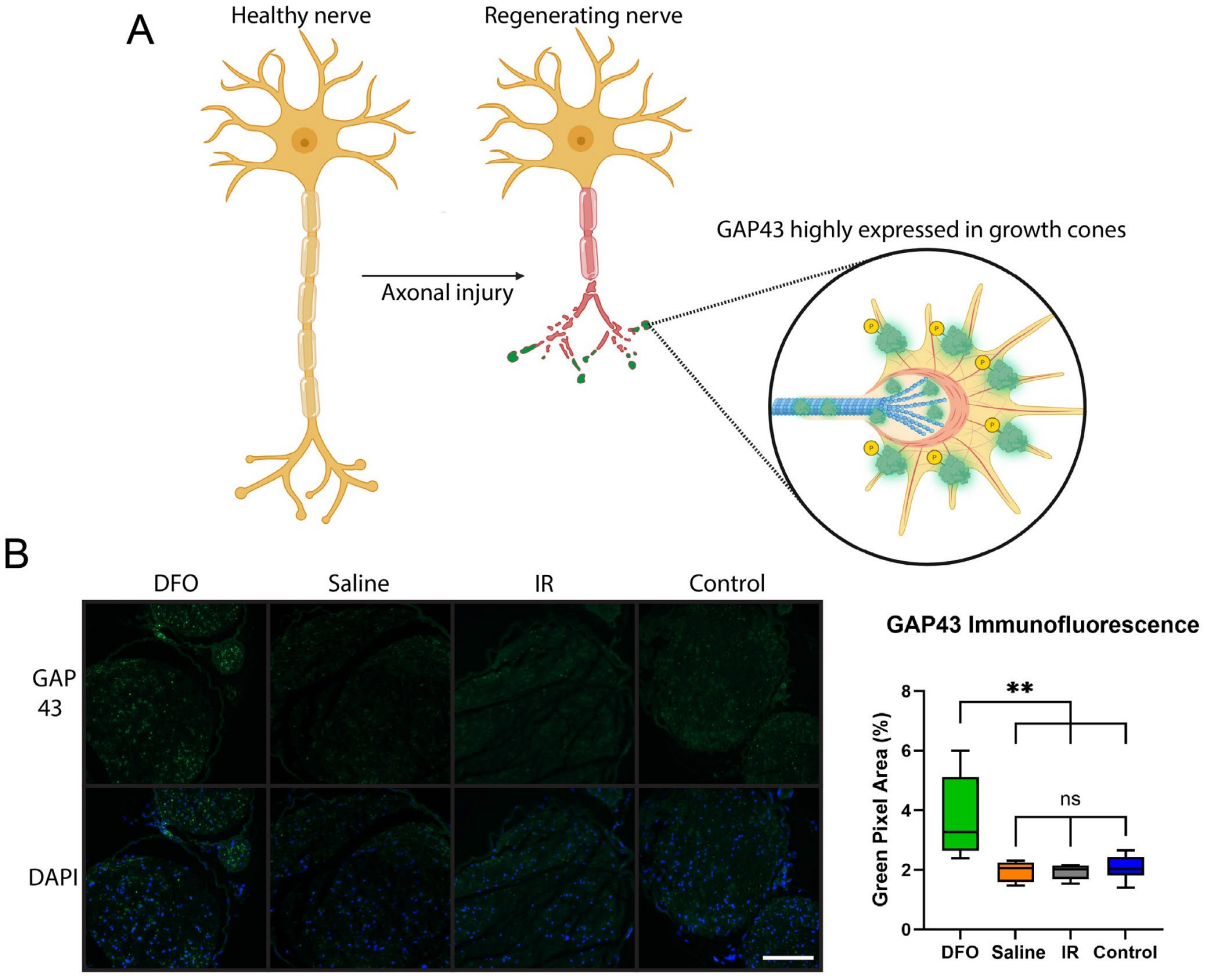
Growth‐Associated Protein 43 function and immunofluorescence. (A) Growth‐Associated Protein 43 (GAP43) is a neuron‐specific phosphoprotein involved in axonal growth and synaptic plasticity. Following axonal injury, its expression is upregulated to aid in peripheral nerve regeneration. It is trafficked to axonal growth cones and synaptic terminals where its phosphorylation acts as a molecular switch to influence Actin dynamics and membrane interactions. (B) GAP43 staining revealed an increase in axonal regeneration in the DFO group only. Results are displayed as raw percentage comparisons, not normalized to contralateral healthy limbs like all previous metrics. (Mean green pixel area was 3.76% for DFO, 1.95% for Saline, 1.94% for IR and 2.09% for Control nerves (**p = 0.0045 for DFO vs. Saline, **p = 0.0043 for DFO vs. IR and **p = 0.0037 for DFO vs. Control, no significant differences were found between Saline, IR or Control nerves)). Microscopy scale bar represents 100 μm. Box and whisker plots with line at medians, whiskers at minimum and maximum values.

Right hindleg irradiated sciatic nerves as well as left hindleg normal sciatic nerves were harvested from each mouse at the conclusion of the experiment. These tissues were collected immediately following euthanasia and placed in ice‐cold PBS. Tissue was fixed in 4% paraformaldehyde for 16 h before graded EtOH dehydration, xylene clearing and paraffinization. Nerves were embedded into paraffin blocks for perpendicular microtome sectioning at 5 μm. To compare myelination, slides were stained with anti‐Myelin Protein Zero (MPZ) primary antibody at a dilution of 1:100 (#57518, Cell Signalling Technology, Danvers, MA, USA) followed by an Alexa‐594 goat‐anti‐rabbit secondary antibody at a dilution of 1:200 (#A‐11012, Thermo‐Fisher Scientific, Waltham, MA, USA). MPZ is an extracellular structural protein involved in myelin lamination, with its expression correlating to healthy, functional nerves [34, 35] (Figure 5A). Similarly, to quantify axon regeneration, slides were stained with anti‐Growth‐Associated Protein 43 (GAP43) primary antibody at a dilution of 1:100 (#5307, Cell Signalling Technology, Danvers, MA, USA) followed by an Alexa‐488 goat‐anti‐rabbit secondary antibody at a dilution of 1:200 (#A‐11008, Thermo‐Fisher Scientific, Waltham, MA, USA). GAP43 is highly expressed at regenerating growth cones and minimally expressed in healthy axons, thus is a common marker for neural regeneration [36, 37] (Figure 6A). Images were obtained at 20× magnification using a Leica DMI4000 B inverted microscope (Leica Microsystems, Wetzlar, Germany). Image scale bars represent 100 μm.

MPZ staining patchiness representing varying intensity within each nerve was observed, so demyelination was better characterized by mean staining intensity rather than counting myelinated versus demyelinated axons. In fact, disruptions in the red fluorescing areas in the images were primarily attributable to histological fixation artefacts/tissue separation rather than fulminant demyelination. An ImageJ (NIH, Bethesda, Maryland, USA) quantification process was used that binarized images and measured average red pixel intensity within a region of interest overlaid onto the nerve to avoid blank background or separated tissue areas. All images were taken with identical laser and exposure settings. Treatment group red pixel intensities were normalized to their respective contralateral control nerve pixel intensities, and the means of these ratios were compared between groups. A similar ImageJ process was performed for green pixel area; though, only the DFO treated nerves showed reliable GAP43 staining above tissue background so means were not normalized to contralateral hindlimbs given unreliable denominators approaching zero. Instead, contralateral non‐irradiated nerves (Control group) were batched and their green pixel area means were compared to the treatment groups means directly. Thus, contralateral hindlimbs acted as each mouse's internal control for all metrics in this study except for GAP43 quantification.

### 2.6 Statistical Analysis

GraphPad Prism 10.6.1 (GraphPad Software, Boston, MA, USA) was used to perform all analyses and generate graphs. Means between groups were compared using two‐way analysis of variance (ANOVA) with Tukey's multiple comparison tests at specific time points. Significance was determined based on a *p‐value < 0.05. Error bars on functional metrics' graphs represent standard deviations. Error bars on immunofluorescence box and whisker plots represent minimum and maximum values.

## 3 Results

### 3.1 Functional Metrics

Irradiated hind limbs demonstrated both motor and sensory deficits in the chronic phase of RIPN (4 weeks after XRT completion—thus experiment week 6) compared to contralateral unaffected hindlimbs. There were no significant differences noted between treatment groups until week 8 after DFO and Saline injections were completed. Interestingly, in assessing motor neuron function, the greatest difference between right and left hindlimbs was noted acutely at week 2 immediately following XRT completion, with mean CTS scores at −14.51, −13.83 and −14.66 for DFO, Saline and IR respectively, with no significant difference between groups at this time. Following DFO treatment, however, mean CTS improved relative to IR and Saline groups: −9.72, −12.91, −12.72 for DFO, Saline and IR, respectively (Figure 3B).

Similarly, sensory deficit was noted in the chronic phase of RIPN. Prior to the chronic phase, however, the deficit was preempted by a sharp increase in allodynia behaviours in the acute setting. Relative to unaffected left legs, all irradiated right legs demonstrated increased cold allodynia duration and decreased monofilament weight requirement to induce mechanical allodynia. It was not until the chronic phase of RIPN that we observed a reversal into hyposensitivity for both measures. At week 8, acetone‐induced cold allodynia duration ratios were 0.90, 0.70 and 0.73 for DFO, Saline and IR, respectively (Figure 4B). Von Frey monofilament testing revealed the same trend with heavier weight rated filaments in the RIPN limb but did not achieve statistical significance between groups. At week 8, the ratios of the lowest monofilament weights to evoke withdrawal responses were 1.16 for DFO, 1.50 for Saline and 1.45 for IR (Figure 4C).

### 3.2 Myelination and Axonal Growth

Immunofluorescence staining for MPZ revealed decreased sciatic nerve myelination relative to healthy contralateral nerves following XRT. Deferoxamine treated nerves showed increased myelination ratios: 0.84 for DFO, 0.74 for Saline and 0.72 for IR (Figure 5B). Since functional metrics in the non‐DFO irradiated nerves did not decline between week 6 and week 8, it is more likely that differences in myelination with DFO treatment are due to augmented remyelination rather than a protective effect.

Immunofluorescence for axonal regeneration revealed increased GAP43 staining in the DFO treated nerves only. Mean green pixel area was 3.76% for DFO, 1.95% for Saline, 1.94% for IR and 2.09% for Control nerves (Figure 6B). The increase in GAP43 activity in DFO nerves implies that Wallerian degeneration occurred as there are regenerating axons present, even if this regeneration was not present in the Saline or IR groups. The minimal GAP43 in the other groups was surprising; however, it is possible that only the DFO group had axonal regeneration processes occurring in this chronic phase of RIPN.

## 4 Discussion

Deferoxamine has been used in clinical practice since the 1960's as a systemic medication administered to treat iron overload states such as hemochromatosis. In more recent years with the development of reverse micelle drug delivery technology, our group has applied deferoxamine locally to irradiated skin revealing its benefits to counteract RIF [21, 22, 38, 39]. Local doses are orders of magnitude smaller than clinically tolerable IV doses, with no detectable spread beyond the local area at such small doses [38]. Given DFO's safety profile and the severe lack of treatment options for RIPN, this study presented an exciting natural intersection between our prior radiation injury work and other groups' mechanical injury experiments evaluating DFO's nerve regenerative potential.

### 4.1 Deferoxamine Improves Peripheral Nerve Functional Deficits

Irradiated hindlegs experienced a decrease in toe spread immediately following irradiation. Examining the trends over the experimental timeline, this was very likely attributable to hypersensitivity and pain. Concurrent increases in allodynia to cold acetone evaporation as well as mechanical stimulation were experienced in all mice. In the chronic phase, however, this progressed to hyposensitivity evidenced by shorter allodynia durations and heavier filaments, yet toe spread distances remained less than their contralateral uninjured feet. This reflects the expected constellation of sciatic injury symptoms that we hoped to rescue with DFO, as sensation‐related symptoms seen in human patients in the chronic phase are more commonly hyposensitivity/numbness instead of pain as well. Indeed, DFO treatment led to an almost 3‐point CTS change compared to Saline/IR hindlimbs representing a ~22% improvement, as well as a 17%–20% improvement in hyposensitivity in the acetone test. The Von Frey monofilament test was not statistically significant, but it followed the same trend and approached significance. Though a modest improvement in these metrics, it is an inspiring finding as these are the symptoms that cause a dramatic detriment to patients' quality of life.

### 4.2 Local Iron Chelation Enhances Remyelination and Axonal Regeneration

Implicated in our functional outcomes are large, myelinated motor neurons at the level of the sciatic, tibial and common peroneal nerves. Additionally, sensory tests like acetone cold allodynia duration favour myelinated Aδ fibres for nociceptive transmission over smaller unmyelinated C fibres [40]. Our examination of MPZ as a proxy for nerve sheath/myelin content revealed that irradiation caused a 26%–28% decrease in MPZ intensity, and DFO administration improved this to a 16% deficit. This provides an in vivo correlation to Zhang et al., who found DFO treatment upregulated Schwann Cell viability, migration capability and nerve growth factor expression in isolated dorsal root ganglia Schwann Cells [37]. In terms of axonal regeneration, the only group to show GAP43 expression reliably above background fluorescence was the DFO treated nerves. GAP43, also known as neuromodulin, regulates actin dynamics at axonal growth cones and presynaptic terminals, so is primarily involved with neuronal growth and plasticity processes [41]. It is highly expressed at the leading edges of regenerating axons in particular. As mentioned earlier, its presence in the DFO group shows irradiation caused axonal injury/Wallerian degeneration in addition to the aforementioned Schwann cell injury and demyelination. The minimal expression of GAP43 in uninjured control nerves is expected; however, the other two irradiated groups also displayed the same minimal level despite being injured. This implies that these groups may have suffered permanent axonal damage without regrowth, which again would align with the unchanging functional test measurements between week 6 and week 8. Ultimately, the Control nerves and Saline/IR nerves had different reasons to express minimal GAP43. DFO's proposed impact on axonal regeneration is twofold: first, the increase in nerve growth factors by isolated Schwann cells, and second, the local angiogenic/antioxidant properties that promote a healing‐centric local milieu by increasing blood flow and mitigating ROS‐associated damage.

### 4.3 Clinical Applications of Deferoxamine for Radiation‐Induced Peripheral Neuropathy

Despite DFO's original use as an IV treatment for systemic iron‐overload states, there is high potential for clinical translatability of DFO for many other conditions now that its cellular and molecular mechanisms are better understood. This study lies at a crossroads between our group's use of DFO for dermal RIF and other nerve injury literature that explores DFO for its regenerative potential. The neuroprotective community has shown interest in DFO as it pertains to central nervous system pathologies; however, several notable studies have also used DFO as a treatment for peripheral nerve injury [42, 43]. Li et al. found that intraoperative DFO application at the time of nerve decompression accelerated axonal regeneration by GAP43 expression timing, as well as decreased malondialdehyde as a quantifier of ischemia/reperfusion insult [27]. Likewise, Dong et al. used DFO as an adjunct to their regenerative scaffold and created a favourable microenvironment for regenerating peripheral nerves by promoting microvascular ingrowth and decreasing detrimental inflammatory processes [28]. These are injuries which occurred on more acute timelines relative to the insidious nature of RIPN, and DFO was shown to be helpful in these settings as well. With increasing understanding of the pathophysiology surrounding different nerve injury types (diabetic/chemotherapy neuropathy, compression, crush, traction, transection, etc.), we suspect local DFO may prove itself to be useful in many more contexts.

Compared to other side effects of XRT like radiation dermatitis that can affect up to 95% of all patients who undergo irradiation, clinically significant RIPN develops in a much smaller percentage and follows an unpredictable course. Therefore, despite DFO's favourable safety profile there may be limited clinical utility in prophylactic administration. Patients presenting with RIPN typically experience local pain combined with distal paresthesias or weakness in the acute setting due to early inflammation and edema, but this resolves spontaneously in the majority of cases. In the chronic setting months to years after XRT, however, prolonged inflammation leads to RIF of the nerves and surrounding tissues which is largely irreversible. Patients experience progressive paresthesia, weakness, muscular atrophy and sometimes pain distal to the irradiated site. It is these patients experiencing chronic symptoms who may benefit from DFO administration, as all current nonsurgical treatment is aimed at symptomatic management (e.g., physical therapy for musculoskeletal strength and mobility or pharmacotherapy for pain). The objective for surgical treatment is the removal of compressive RIF‐afflicted perineural structures (neurolysis) to improve severe symptoms. Unfortunately, even surgical outcomes are bleak when sensorimotor deficits are involved, with < 20% of brachial plexus RIPN patients reporting improvement in their limb function [44]. On the other hand, 53% of those patients did report improvements in their pain. Given this disheartening clinical environment surrounding the treatment of RIPN, there exists a void that a locally administered tissue‐conditioning therapy can fill. Either as an injection prior to considering surgery or as an intra‐operative topical adjunct, DFO would be relatively easy to administer into the current treatment workflow (of which there is no established algorithm to adhere to). As DFO is a bulky molecule with a relatively short half‐life on the order of hours, this would also create interest in an extended‐release mechanism like a hydrogel or encapsulated formulation that can be injected under imaging guidance or directly applied if the operating room [45].

Ultimately, this murine study suggests DFO has utility as a treatment for RIPN from a functional and histological viewpoint. These findings are encouraging as this condition currently has no validated disease modifying treatments on the market. Further study is needed to examine the extent of radiation injury at the level of the nerve versus the surrounding soft tissues. This will allow us to investigate whether DFO's effectiveness is at the nerve itself or in reversing the compressive effect of surrounding soft tissue fibroatrophic changes. Given the multi‐pronged mechanism of action, we suspect some degree of both. Additionally, refinement of the RIPN murine model is necessary. We were successful in creating functional deficit with our prior dermal fibrosis 30 Gy fractionated protocol; however, greater initial functional detriment or dose optimization may increase the treatment effect size [41]. As expected, our findings in a murine model differ in time course than rat studies; however, many of the same measurement techniques remain applicable [46]. There will undoubtedly be further differences encountered in large animals and humans. Nevertheless, local deferoxamine administration holds promise as a safe, readily available treatment for radiation‐induced peripheral neuropathy, for which current options are limited.

## 5 Conclusion

Mirroring studies that have shown deferoxamine's benefit in mechanical nerve injury models, as well as the conditioning of dermal radiation‐induced fibrosis, we show that local iron chelation is a promising treatment for radiation‐induced peripheral neuropathy. This study reveals that deferoxamine's benefit is in part due to augmented nerve regeneration, remyelination, and possibly the conditioning of the surrounding soft tissue, which can have a compressive effect on nerves in irradiated fields.

## Author Contributions

**Alexander Z. Fazilat:** conceptualization, investigation, writing – original draft, formal analysis, data curation. **Christopher V. Lavin:** conceptualization, investigation, writing – original draft, formal analysis, data curation. **Sriya Nemani:** data curation. **Carter B. Kendig:** data curation, formal analysis. **Michelle Griffin:** resources, writing – review and editing. **Palca Shibale:** data curation. **Derrick C. Wan:** supervision, resources, writing – review and editing. **Kelly X. Huang:** data curation. **Jennifer B. Parker:** data curation. **Naga A. R. Ailury:** data curation. **Michael T. Longaker:** resources, writing – review and editing, supervision. **Caleb Valencia:** data curation. **Arash Momeni:** resources, writing – review and editing, supervision.

## Funding

This research was supported by the Center for Dental, Oral and Craniofacial Tissue and Organ Regeneration (C‐DOCTOR grant U24DE026914).

## Conflicts of Interest

Dr. Derrick Wan and Dr. Michael Longaker hold a patent for the use of deferoxamine in conditioning irradiated tissue.

## Data Availability

The data that support the findings of this study are available from the corresponding author upon reasonable request.
